# Feeling Safe With Hypnosis: Eliciting Positive Feelings During a Special State of Consciousness

**DOI:** 10.3389/fpsyg.2022.917139

**Published:** 2022-06-02

**Authors:** Barbara Schmidt

**Affiliations:** ^1^Institute for Psychosocial Medicine, Psychotherapy and Psychooncology, Jena University Hospital, Jena, Germany; ^2^Institute for Clinical Psychology, University of Jena, Jena, Germany

**Keywords:** hypnosis, anxiety, arousal, decision-making, intensive care unit, delay discounting, risk behavior, safety

## Abstract

Our state of consciousness is crucial for our ability to follow suggestions. Suggestions in turn are a powerful tool to induce positive emotional states. In my research, I suggest positive feelings of safety during hypnosis. This is a positive emotional state of low arousal and low anxiety. Both arousal and anxiety affect our decision-making. However, when we feel safe due to hypnotic suggestions of safety, we do not act riskier. Instead, EEG brain activity shows that monetary rewards get less important and delayed rewards are less devalued compared to immediate rewards when we feel safe. These results open promising perspectives for the use of hypnosis to reduce impulsive behavior, for example, in substance abuse. Therapeutic suggestions of safety even work in highly stressful environments like the intensive care unit. I showed that patients tolerate non-invasive ventilation much better when they get the suggestion to feel safe. The effects of positive therapeutic suggestions delivered during hypnosis even persist over time. Post-hypnotic suggestions are associations between a certain emotional state and a trigger that elicits this emotional state after hypnosis is over. I showed that post-hypnotic suggestions of safety are effective weeks after the therapeutic session. Therefore, I present a therapeutic technique that uses a special state of consciousness, hypnosis, to induce positive emotional states. The effects of this technique are very strong and long lasting. My goal is to provide scientific evidence for the use of hypnotherapeutic techniques to increase the number of people who apply and profit from them.

## Hypnosis as a Special State of Consciousness

Different states of consciousness are a normal part of our life. When we sleep, we are in a certain state of consciousness. When we are in the middle of doing something that we really like, we can also get into a very special state of consciousness called trance or flow. A trance or flow state of consciousness enables effortless performance and is associated with positive feelings. Musicians often perceive this state of consciousness as an optimal balance between their skills and the challenge they are facing, an experience which is related to high performance and incompatible with stage anxiety (Cohen and Bodner, 2019). Professional athletes experiencing flow report to have a clear idea of their goals and get unambiguous feedback, which in turn predicts high satisfaction with life (Habe et al., 2019). High performance is often accompanied by a trance state where it is possible to show your optimal performance, because you are totally absorbed by the moment and nothing else matters. It is a state of very intense focus on the one thing that matters while ignoring all other irrelevant stimuli. Some even use potentially distracting stimuli to get deeper into their optimal state of performance, a technique described by one of the most important pioneers in hypnosis, Milton Erickson (1959).

When we elicit this trance state *via* a hypnosis induction, we call it hypnosis. The current definition of hypnosis was stated by the APA Division 30 in the year 2015. The division defines hypnosis as follows: “A state of consciousness involving focused attention and reduced peripheral awareness characterized by an enhanced capacity for response to suggestion” (Elkins et al., 2015). Suggestions are contents that the hypnotist says. An example for a suggestion is: “The longer you look at this point in front of you, the heavier get your eyelids so that you want to close them.” The hypnotized person can then accept this suggestion and close his or her eyes. Following a suggestion feels like an automatic process instead of a conscious decision. It feels natural to follow the suggestion and close your eyes. The ability to follow suggestions is dependent on the rapport between the hypnotist and the hypnotized person, describing a positive therapeutic relationship of trust and responsibility. Suggestibility is usually measured with the Harvard Group Scale of Hypnotic Susceptibility (HGSHS; Shor and Orne, 1963). This is a group test consisting of a hypnosis induction and 12 suggestions. Participants indicate on a questionnaire if they followed the suggestions, resulting in a score from 0 for very low suggestibility to 12 for very high suggestibility.

Hypnosis as a special state of consciousness has long been fascinating scientists. Ivan Pavlov reported that some of his dogs were in a trance-like state after his experimental procedures of classical conditioning (Pavlov and Petrova, 1934). Hans Jürgen Eysenck studied the differences between primary and secondary suggestibility, which describes the ability to follow suggestions (Eysenck and Furneaux, 1945). After the invention of the electroencephalogram (EEG) in Jena (Berger, 1929), the famous Berger effect was investigated with hypnosis. In Berger’s original experimental setup, the participant was opening and closing his eyes while Berger measured the participant’s EEG alpha activity. Alpha activity was only visible in the EEG signal when the participant’s eyes were closed and blocked when eyes were open. In the hypnosis study, the participant was suggested during hypnosis that he was blind or that he can see. When the participant was suggested to be blind, EEG alpha activity was visible, even though the eyes were open (Loomis et al., 1936). This observation shows that sensory processes can be altered with hypnotic suggestions.

## Blocking Sensory Perception Under Hypnosis

**FIGURE 1:**
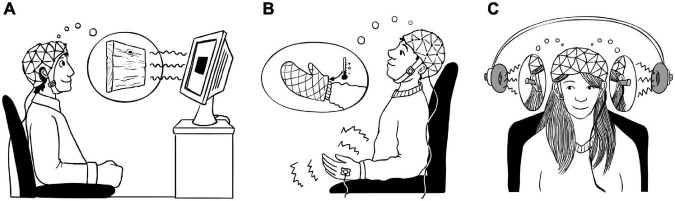
Illustration of the three EEG studies using hypnotic suggestions to block sensory processing. **(A)** Participants had to count rare visual stimuli on the screen. In the hypnosis condition, they were suggested that a wooden board is blocking their vision on the screen, so they cannot see the visual stimuli. **(B)** Participants received electrical pain stimuli on their hand. In the hypnosis condition, they were suggested that a cooling and numbing glove is covering their hand, so they cannot feel the stimuli. **(C)** Participants had to press a button when they heard a rare sound. In the hypnosis condition, they were suggested that earplugs keep them from hearing the sounds.

In three separate EEG studies, I blocked sensory perception of visual, pain, and auditory stimuli using hypnotic suggestions of a wooden board in front of participants’ eyes, a cooling and numbing glove on participants’ hands and earplugs in participants’ ears, respectively (Figure 1; Schmidt et al., 2017b; Franz et al., 2020, 2021). We used a sensory paradigm that is very common in EEG research with frequent and rare stimuli, called oddball paradigm. In the visual study, participants had to count the rare stimuli on the screen, which were colored squares in my study. Brain responses usually show a very clear response to the rare to be attended stimuli in the so called P3 response. That is a positive voltage change about 300 milliseconds after the stimulus was presented. I had three groups of participants in this study, selected according to their HGSHS suggestibility scores (Shor and Orne, 1963). I had 20 low suggestible, 20 middle suggestible and 20 high suggestible participants. All participants played the oddball task twice in counterbalanced order. Once with hypnosis and the suggestion that a wooden board is blocking their vision (Figure 1A), once in a control condition without hypnosis. In both conditions, participants saw the stimuli of the oddball task on the screen, presented one at a time, and counted the rare colored squares. While participants sat in the EEG chamber, I checked that their eyes were open all the time. The results show that participants were not able to correctly count the rare stimuli in the hypnosis condition, while they showed almost perfect counting performance in the control condition. Participants’ brain responses showed that the visual stimuli were still perceived in the hypnosis condition, indicated by early event-related EEG components. But the target P3 component was massively reduced in the hypnosis condition. The smaller the P3 amplitude was, the more reduced was participants’ counting performance and the more vivid was their experience of the wooden board in front of their eyes in the hypnosis condition. The effect was strongest for high suggestible participants. The results show the neuronal dissociation between perceiving the visual objects on the screen and attending them in order to count them, reflected in early and late event-related EEG signals. In a subsequent analysis, we found that this effect was driven by a top-down modulation, reflected in reduced directed information flow from parietal attentional to frontal executive sources during processing of target stimuli (Franz et al., 2021).

We obtained similar results in the two subsequent EEG studies where I blocked pain and auditory processing with hypnotic suggestions. In the pain study, I blocked participants’ pain processing *via* the suggestion of a glove that keeps the stimulated hand from feeling pain (Figure 1B) similar to the suggestion by Rainville et al. (1997). In the auditory study, I blocked participants’ auditory processing *via* the suggestion of earplugs (Franz et al., 2020). In these studies, I included additional control conditions of attention distraction and simulation of hypnosis. In the auditory study, we used an auditory oddball paradigm where participants are presented frequent and rare sounds. Participants had to press a button when they heard the rare target sound. In participants’ EEG, we focused again on the P3 component to the rare target sounds. We found a significant reduction in P3 amplitudes in the hypnosis condition compared to the control condition. Participants also pressed the button to the target sound significantly less often and perceived the sounds as less loud in the hypnosis condition compared to the control condition (Franz et al., 2020). Taken together, the results of the three sensory blockade studies reveal that hypnotic suggestions are a powerful tool to modify sensory processes in the brain, especially processes that are associated with attention control and stimulus evaluation like the P3 amplitude.

## Hypnosis, Arousal, and Decision-Making

The state of hypnosis is not only characterized by an enhanced ability to follow suggestions, but also by low arousal. When you are in hypnosis, you are relaxed. Therefore, hypnosis was a valuable tool for the development of systematic desensitization as described by Wolpe et al. (1973). In his description of the first standardized technique in psychotherapy, Wolpe uses hypnosis to relax the patient before the patient imagines the objects or situations that he or she is afraid of. The imagination of fear stimuli can be as efficient as real fear stimuli, as revealed by a recent fear conditioning study (Mueller et al., 2019). In this study, participants imagined stepping into a thumbtack when a certain visual stimulus appeared on the screen. Participants developed a conditioned fear response like in previous fear conditioning studies that used real instead of imagined stimuli. The study by Mueller et al. (2019) provides further evidence for the effectiveness of imagination. To reduce fear responses, participants can use hypnosis to get relaxed and then imagine the previously fear-eliciting stimulus. As Wolpe et al. (1973) report, this is a very effective method to reduce anxiety.

Reducing participants’ arousal typically affects their decision-making behavior. I showed that participants who have generally lower arousal, indicated by a low resting heart rate, acted riskier in a risk game than participants with higher arousal (Schmidt et al., 2013). When participants’ state arousal was increased after riding the bike for 10 min on a bicycle home-trainer, they tended to be less risky (Schmidt et al., 2013). Being aroused often goes along with being anxious. I found that more anxious participants acted less risky in a risk game and showed higher frontal midline theta power than less anxious participants (Schmidt et al., 2018). When participants wore a bike helmet, they showed lower frontal midline theta power, but did not generally act riskier in a risk game compared to participants without bike helmet (Schmidt et al., 2019). These studies show that lower arousal is associated with less anxiety and riskier behavior. Reducing arousal with hypnosis might therefore also reduce anxiety and affect decision-making.

## Feeling Safe With Hypnosis

One of the most prominent techniques in hypnotherapy is to suggest participants that they are at a safe place. The need for safety was stated as one of our most basic needs by Abraham Maslow (1943). In his seminal publication that resulted in his famous pyramid showing the hierarchy of needs, he states: “Practically everything looks less important than safety (even sometimes the physiological needs which being satisfied, are now underestimated). A man, in this state, if it is extreme enough and chronic enough, may be characterized as living almost for safety alone” (Maslow, 1943). The safe place hypnosis technique uses the suggestion that the hypnotized person is at his or her personal safe place (Arntz, 2011). The hypnotized person is free to choose his or her own imaginations, the hypnotist only offers suggestions. One example would be: “Be curious what you can see, hear and smell at your safe place. Feel the place in your body. Where is this feeling most intense? Focus on this part of your body and make the feeling grow even stronger. It radiates through your whole body” (Schmidt et al., 2020).

I developed a standardized safe place suggestion and measured brain responses and behavior of participants in a risk game. For this study, I only invited highly suggestible participants that were again pre-tested with the HGSHS (Shor and Orne, 1963). Participants played a risk game twice in two conditions while recording their EEG brain responses (Schmidt et al., 2020). In the hypnosis and safety condition, I hypnotized participants and suggested them to be at their own safe place. Then, they played the risk game. In the control condition, participants played the risk game without hypnosis. To understand the results of this study, it is important to know that monetary rewards elicit a P3 response. Higher monetary rewards are reflected in higher P3 responses (Begleiter et al., 1983). The same is true for other rewarding stimuli. For example, smokers respond with a strong P3 amplitude to images related to smoking compared to other images. In my study, I used monetary rewards as incentives. My results show that participants showed significantly lower P3 amplitudes to all monetary rewards, large or small, when they felt safe in the hypnosis condition compared to the control condition. Excitingly, similarly reduced P3 amplitudes were reported in smokers who no longer smoke (Littel and Franken, 2007). While smokers in the study had to be abstinent for a long time to stop having strong P3 responses to the smoke pictures, in my study only one hypnosis session with safety suggestion was sufficient. Imagining a safe place may be a way to stop having strong responses to reward stimuli. This could aid in the treatment of addictive disorders. If people stop reacting strongly to stimulants to which they are addicted, they will find it easier to give up consuming these stimulants. Importantly, risk behavior did not change when participants felt safe during hypnosis. Therefore, there is no contraindication to use suggestions of safety in patients suffering from addiction.

In a second paradigm, I investigated another phenomenon, the devaluation of future rewards, also called delay discounting (Schmidt and Holroyd, 2021). You are probably familiar with the famous marshmallow task by Mischel et al. (1988). A preschool-aged child is presented with the task of either eating one marshmallow now or waiting to get two marshmallows. Children who were able to wait for the second marshmallow showed better social and academic performance later (Mischel et al., 1988). Thus, the ability to wait for later rewards is desirable. In this context, children’s decisions to wait for rewards also depends on how much they trust their environment (Mahrer, 1956; Kidd et al., 2013). I therefore hypothesized that individuals who feel safe would be more willing to wait. In the delayed gratification game that I used in my EEG study (Schmidt and Holroyd, 2021), participants could win immediate monetary rewards and rewards that were paid 6 months later. The EEG brain activity shows more positive deflections after an immediate reward than after a delayed reward. The difference between the deflection of the EEG signal to immediate and delayed rewards is called reward positivity. If this difference is large, our brain makes a strong distinction between immediate and delayed rewards, and it is difficult to wait. However, if this difference is small, we will find it easier to wait. I found almost no difference between EEG brain responses to immediate and delayed rewards in participants who felt safe. In contrast, they showed strong EEG differences in the control condition. When I compare the results of this study with the results of an earlier study using the same paradigm (Schmidt et al., 2017a), it becomes even clearer how exciting these findings are. In the earlier study, I compared two groups of participants. One group was low impulsive and high self-controlled, and the other group was high impulsive and low self-controlled. Participants in the low impulsivity group showed a comparably small difference between immediate and delayed rewards as did the participants in my current study (Schmidt and Holroyd, 2021) when they felt safe during hypnosis. And this was after a single hypnosis session with the suggestion of being at a safe place. It might therefore be possible to make participants less impulsive and more controlled by suggesting them to be at a safe place. This, in turn, could make it easier for them to wait for future rewards. Forgoing immediate rewards in favor of future rewards requires a high degree of self-control and confidence that waiting will pay off. This skill is essential in coping with individual problems such as substance addiction and obesity, as well as global problems such as climate change and a pandemic.

After these encouraging results, I decided to link the feeling of safety to a post-hypnotic trigger (Böhmer and Schmidt, 2022). I wanted to investigate whether this could trigger the feeling of being in a safe place in the long term without the need for another therapeutic session. In order to establish a safety trigger, I used a white sheet of paper on which participants under hypnosis wrote the letter S for safety. I suggested the participants that the feeling of safety was stored in the piece of paper. Every time they looked at this piece of paper, folded it up and put it into their pocket, the feeling of safety should automatically reappear. In the EEG study, everything was similar to what I described earlier (Schmidt et al., 2020; Schmidt and Holroyd, 2021), except that I ended the hypnotic state before playing the risk game in the safety condition. Participants were then given the S paper or a neutral paper with the letter K for control on it. They again played the risk game twice, once with the post-hypnotic suggestion of safety and once with the neutral paper as the control condition. Again, participants’ risk behavior did not differ in both conditions. We analyzed the difference between the EEG brain responses to high and low monetary rewards. A large difference here is an indicator of strong reward sensitivity. The brain then responds strongly to rewards. We found that participants’ EEG brain responses in the safety condition made almost no difference between high and low monetary rewards, while in the control condition they made a very strong distinction between high and low rewards. The reduced responsiveness for reward stimuli indicates a state of satisfaction and satiation. Such a state is very helpful in treating individuals who are otherwise very responsive to reward stimuli such as individuals suffering from substance abuse. We also asked participants weeks after the initial experimental session if the post-hypnotic safety trigger still worked. Participants indicated that the S piece of paper still elicited a feeling of safety, showing the long-lasting effects of post-hypnotic suggestions.

## Feeling Safe in the Intensive Care Unit With Hypnotic Suggestions of Safety

**FIGURE 2:**
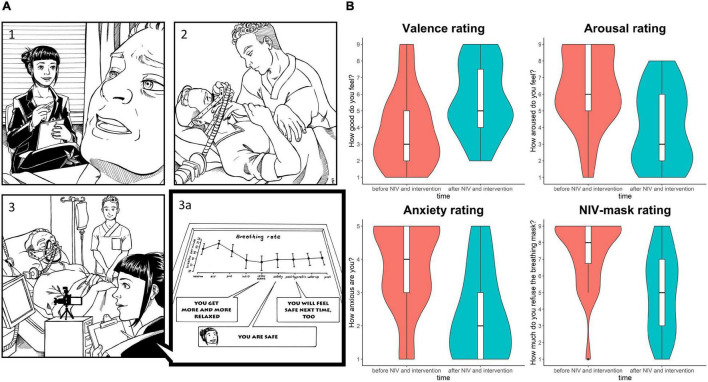
**(A)** Illustration of the procedure of the intensive care study. The psychologist asked the patient about his current emotional state. Then, a member of the medical staff puts on the NIV breathing mask. During the ventilation session, the psychologist accompanies the patient with therapeutic suggestions of safety. The vital sign monitor shows that breathing rate was reduced during safe place suggestions. **(B)** Results of ratings before and after NIV sessions accompanied with safe place suggestions showing better mood, less arousal and anxiety, and a more positive evaluation of the NIV mask after the intervention.

To prove the effectiveness of a therapeutic suggestion, it is important to show that it works in naturally occurring situations. I therefore did a study in which we used the safe place method inside the intensive care unit (Schmidt et al., 2021). In this study, my master’s student Jana Schneider accompanied patients who have strong fear of non-invasive ventilation. Non-invasive ventilation can cause feelings of suffocation when trying to breathe against the machine, even though ventilation is intended to ensure that the patient’s body is optimally supplied with oxygen. Regular ventilation sessions in the intensive care unit last about 15 min. We accompanied the patients during one of those ventilation sessions. The ventilation and the suggestion of the safe place thus took place simultaneously. We included only patients who were awake and able to provide information about their current state. Before and after the ventilation session, we asked patients how anxious they were, how aroused they were, and how well they generally felt. During ventilation, we recorded the patient’s vital signs monitor. This allowed us to analyze exactly how the patients’ bodies responded to the safe place suggestion. We found that the respiratory rate was reduced as a sign of relaxation during the safe place suggestion and the heart rate also calmed down. After the intervention, patients reported feeling less anxious, less aroused, and generally feeling better. They also rated the breathing mask itself as less negative. Figure 2 shows the procedure and results of this study.

## Conclusion

In my studies I showed that the suggestion of a safe place is very effective both in the EEG laboratory and in the intensive care unit during challenging medical procedures. The effects were particularly large in the intensive care unit, where we assume a naturally occurring trance state that contributes to the effectiveness of the suggestions. From my EEG studies, I can draw conclusion about the effect of safety suggestion under hypnosis and as a post-hypnotic suggestion. Immediately after hypnosis, subjects felt safer than with post-hypnotic suggestion, with the effectiveness of post-hypnotic suggestion lasting for weeks. In summary, the studies confirm the high efficacy and good applicability of the safe place therapeutic technique. It is my sincere wish that through my research I will contribute to the even more widespread use of this technique and help even more people to turn fear into safety.

## Author Contributions

BS wrote the manuscript, contributed to the article, and approved the submitted version.

## Conflict of Interest

The author declares that the research was conducted in the absence of any commercial or financial relationships that could be construed as a potential conflict of interest.

## Publisher’s Note

All claims expressed in this article are solely those of the authors and do not necessarily represent those of their affiliated organizations, or those of the publisher, the editors and the reviewers. Any product that may be evaluated in this article, or claim that may be made by its manufacturer, is not guaranteed or endorsed by the publisher.
